# Expression of CD25 antigen on CD34+ cells is an independent predictor of outcome in late-stage MDS patients treated with azacitidine

**DOI:** 10.1038/bcj.2014.9

**Published:** 2014-02-28

**Authors:** P Miltiades, E Lamprianidou, T P Vassilakopoulos, S G Papageorgiou, A G Galanopoulos, S Vakalopoulou, V Garypidou, M Papaioannou, E Hadjiharissi, V Pappa, H A Papadaki, E Spanoudakis, K Tsatalas, I Kotsianidis

**Affiliations:** 1 Department of Hematology, Democritus University of Thrace, Alexandroupolis, Greece; 2 Department of Hematology, Laikon General Hospital, National and Kapodistrian University of Athens, Athens, Greece; 3 Second Department of Internal Medicine, Hematology Unit, University General Hospital Atticon, Athens, Greece; 4 Department of Clinical Hematology, G. Gennimatas Hospital, Athens, Greece; 5 Second Propedeutic Department of Internal Medicine, Aristotle University of Thessaloniki, Hippokration Hospital, Thessaloniki, Greece; 6 Department of Haematology, Aristotelion University of Thessaloniki, AHEPA Hospital, Thessaloniki, Greece; 7 Department of Hematology, Theageneion Hosp. of Thessaloniki, Thessaloniki, Greece; 8 Department of Hematology, University Hospital of Heraklion, Heraklion, Greece

The expression of CD25 antigen, the α-chain of the interleukin-2 receptor (IL-2Rα), has been repeatedly shown to be associated with poor outcome in adults with either acute myelogenous (AML)^1, 2, 3^ or lymphoblastic leukemia.^4, 5^ To explain this finding mechanistically, it has been hypothesized that CD25^+^ AML blasts are enriched in chemoresistant leukemia stem cells (LSCs), as they bear an LSC-like molecular signature,^2^ are quiescent and can establish AML in xenograft models.^6^

Blasts from patients with myelodysplastic syndrome-related AML (AML-MDS) appear to express CD25 more frequently compared with *de novo* AML patients,^7^ but although late-stage MDS is typically chemoresistant, the prognostic relevance of CD25 has not been investigated yet. On the basis of the above, and as there is paucity of a serviceable biomarker of outcome in MDS patients treated with azacitidine, we addressed the prognostic impact of CD25 expression in a cohort of 61 patients with IPSS intermediate-2/high-risk MDS and chronic myelomonocytic leukemia-2 managed between June 2009 to September 2013 at eight Greek institutions in a non-clinical trial setting.

Following Institutional Review Board approval, bone marrow samples were obtained before and 15 days (D15) after treatment initiation. All patients had an Eastern Cooperative Oncology Group (ECOG) performance status of 0–2 and received azacitidine in a non-clinical trial setting at an initial dose of 75 mg/m^2^ SC for 7 days on 28-day cycles. Granulocyte colony-stimulating factors and erythropoiesis-stimulating agents were used at the discretion of the treating doctor. Response to therapy was evaluated using the International Working Group (IWG) Response Criteria for MDS.^8^ CD25 expression was assessed by 4-color flow cytometry on total CD34^+^ blasts, the compartment of committed progenitors (Lin^−^CD38^+^CD34^+^) and LSCs (Lin^−^CD38^−^CD34^+^, Supplementary Materials and Methods). Positivity was defined as a CD25 expression of ⩾20% above the isotype control. Statistical comparisons were performed by using *χ*^2^, Mann–Whitney *U*-test, and repeated measures analysis of variance or paired *t*-test as appropriate, and survival analysis with Kaplan–Meier and log-rank test. Overall survival (OS) was defined as the time from azacitidine initiation to death from any cause and event-free survival (EFS) as the time from azacitidine initiation to disease progression, relapse or death. Multivariate survival analysis was based on Cox's proportional hazards model using a backward stepwise selection procedure with entry and removal criteria of *P*=0.05 and *P*=0.10, respectively.

The cohorts of CD25^−^ and CD25^+^ patients were well balanced for most known predictive factors and characteristics, except sex (Supplementary Table S1). The median follow-up time from the onset of azacitidine for all patients was 37.3 months and the median number of completed cycles was 5 (1–37). No difference in overall response rate was observed in relation to CD25 status (*P*=0.4), although the complete response (CR) rate in CD25^−^ patients (33%) was two times higher compared with CD25^+^ subjects (16%). In univariate analysis, only CD25 status and heavy transfusion requirements, defined as ⩾4 units of red blood cells (RBC) per month, were predictors for outcome (Figures 1a and b, Table 1 and Supplementary Table S2). We also applied the prognostic score proposed by Itzykson *et al.*^9^ in 58 evaluable patients. Three groups with different outcome were identified with marginally statistically significant median OS (*P*=0.08) and EFS (*P*=0.06), potentially because only 4 (7%) patients were stratified as low risk (data not shown), indicating, however, that despite its limited size, our patient sample was representative in comparison with the existing literature.

Compared with CD25^+^ individuals, the CD25^−^ ones enjoyed significantly longer median OS (16.2 vs 8.8 months, respectively, *P*=0.026) and median EFS (13.6 vs 6.7 months, *P*=0.023). Also, heavily transfused patients had significantly worse OS (7.2 vs 11 months, *P*=0.029) and EFS (6.3 vs 10.4 months, *P*=0.028) compared with those requiring <4 units of RBC per month. Multivariate analysis confirmed the independent prognostic power of CD25 and transfusion requirements for both OS and EFS (*P*=0.01 and *P*=0.008 for both CD25 and transfusion requirements, Table 1 and Supplementary Table S2). A simple scoring system was formed based on these two parameters by attributing one point to each of CD25 positivity and transfusions of ⩾4 RBC units/month (Figure 1c). Patients were stratified in three groups with significantly different median OS (*P*=0.002) and EFS (*P*=0.001), namely, low risk (score 0, OS:18.8 months and EFS:16.2 months, *n*=23), intermediate risk (score 1, OS:10 months and EFS:8.8 months, *n*=30) and high risk (score 2, OS:5.1 months and EFS:4.3 months, *n*=8).

The average expression of CD25 in CD34^+^ blasts of all patients was 21.6±3%. Compared with committed progenitors, LSCs displayed significantly higher CD25 expression (19.4±3% vs 24.1±3.6%, respectively, *P*=0.027, Supplementary Figure S1). We also assessed the kinetics of CD25 expression in 17 patients during azacitidine treatment. On day 15 of the first cycle, CD25 was significantly downregulated in CD34^+^ cells (*P*=0.027), but we weren't able to find any significant correlation of CD25 downregulation with outcome, potentially due to the limited patient number. Interestingly, CD25 was significantly downregulated in the LSC compartment (49.3±7.9% vs 42.7±7.4%, *P*=0.024), but remained stable in committed progenitors of the same patients (33.6±6.6% vs 28.2±5.6%, *P*=0.14), indicating a particular sensitivity of the CD25^+^ subset of LSCs in azacitidine (Supplementary Figure S2). Of note, we observed that despite the marked downregulation of CD25 in CD34^+^ cells, its expression on T cells of the same patient was not affected by azacitidine therapy (Supplementary Figure S3). Although this phenomenon is not readily interpretable, the above differential effect of azacitidine suggests the presence of distinct mechanisms of DNA methylation modulation in these cell types, such as targeting cell-type-specific regulatory elements important for CD25 expression. Alternatively, CD25 may merely represent a surrogate marker of an immature leukemic subpopulation sensitive to hypomethylating agents, which is downregulated via the epigenetic modulation of other unrelated pathways.

Both the revised IPSS^10^ and the GFM scoring system^9^ appear to provide prognostic discrimination in MDS patients treated with azacitidine. However, there is lack of a serviceable, widely accepted biomarker of response and/or outcome that can offer a valid estimation of the expected benefit from azacitidine and help to tailor treatment. Our findings reveal an independent prognostic value of CD25 in such patients. Moreover, we show that a simple prognostic system based on only two parameters, that is, CD25 expression and transfusion dependency, can categorize three groups of patients with significantly different outcome.

The poor outcome of CD25^+^ AML patients has been attributed to the chemoresistant properties of the LSC-like, CD25^+^ myeloblasts, a fact illustrated on the higher rates of both induction failure^2^ and relapse.^1, 3^ By contrast, we observed comparable response rates to azacitidine among CD25-positive and -negative cases, while CD25^+^ myeloblasts were equally reduced on day 15 in both responding and not responding patients (data not shown). This finding is not surprising as the achievement of CR after azacitidine therapy is not an obligate state for better outcome in late-stage MDS. Both the AZA-001 trial and a subsequent analysis of the data argued that all response categories, including stable disease, were associated with a significantly reduced death risk.^11, 12^ In addition, the lower EFS of CD25^+^ patients indicates that the persistence of azacitidine-resistant CD25^+^ LSCs is potentially responsible for the progression or relapse of the disease in these patients. In line with this scenario, it has been recently demonstrated that although the size of the LSC pool correlates with the response to azacitidine, LSCs cannot be eradicated even in responding patients.^13^

The biological role of CD25 expression in AML remains unknown and the effect of IL-2 on the proliferative status of CD25^+^ blasts is also controversial.^1^ CD25 may impart environmental signals, whereas high plasma levels of enzymatically cleaved, soluble CD25 in AML may suppress the antitumor response via competition with the lymphocyte surface CD25 for IL-2 and have long been shown to be associated both with the burden and severity of the disease.^14^

In conclusion, we demonstrate for the first time that CD25 expression status, combined or not with the transfusion requirements, serves as an easy-to-use predictor for outcome in late-stage MDS patients treated with azacitidine, a finding that deserves validation in larger cohorts. Moreover, the differential expression and epigenetic modulation of CD25 in the LSC compartment provide the rationale for the investigation of therapeutic strategies using monoclonal antibody targeting combined with epigenetic agents.^15^

## Figures and Tables

**Figure 1:**
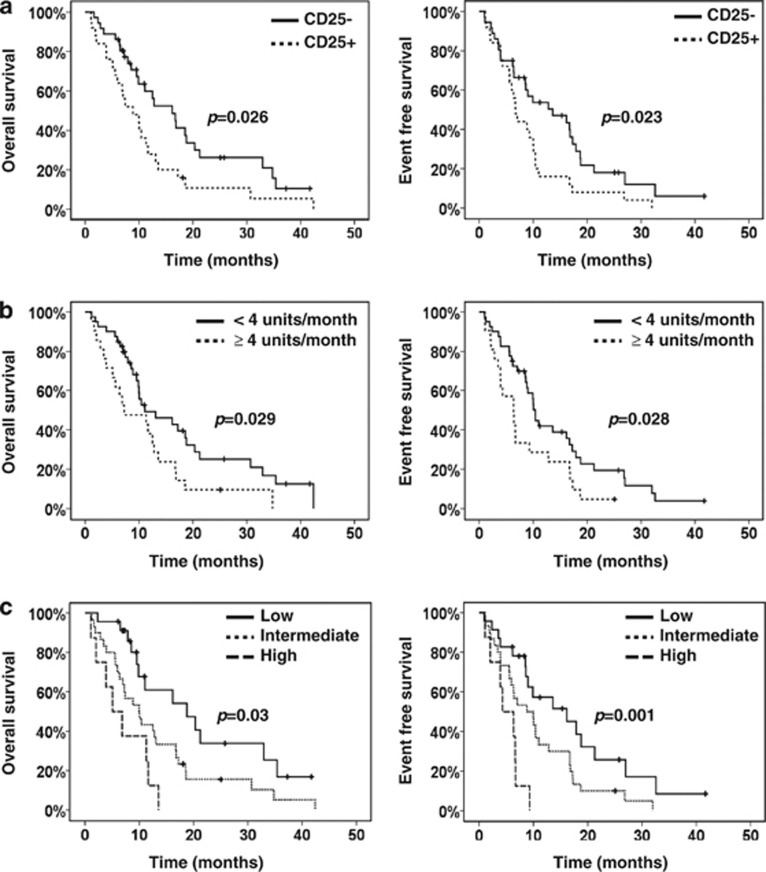
Prognostic parameters for outcome in our MDS cohort. (**a**) OS and EFS in relation to CD25 positivity status. (**b**) OS and EFS in relation to transfusion requirements. (**c**) Prognostic scoring system for outcome based on CD25 positivity (one point) and transfusion requirements of ⩾4 RBC units/month (one point). Kaplan–Meier curves for low (score 0), intermediate (score 1) and high (score 2) risk patients are shown.

**Table 1:** Prognostic factors for overall survival

	*Univariate analysis*		*Multivariate analysis*	
	*Median OS*	P*-value*	*HR (95% CI)*	P*-value*
*Age*		0.73		
>65	10.4			
<65	16.8			
				
*Sex*		0.79		
Male	11.3			
Female	11.1			
				
*CD25 status*		0.026		0.01
Positive (>20%)	8.8		2.15 (1.2–3.9)	
Negative (<20%)	16.2		1	
				
*ANC ( × 10*^*9*^*/l)*		0.47		
⩾1	10.4			
<1	12.5			
				
*Platelets ( × 10*^*9*^*/l)*		0.21		
⩾100	13.1			
<100	10.0			
				
*IPSS*		0.61		
Intermediate-2	12.5			
High	10.4			
				
*WPSS*		0.77		
High	13.1			
Very high	10.4			
				
*IPSS-R*		0.69		
Intermediate	35.4			
High	11.6			
Very high	10.4			
				
*IPSS-R cytogenetic risk*		0.72		
Good	10			
Intermediate	9.5			
Poor	12.8			
Very poor	11.3			
				
*PB blasts*		0.63		
Present	8.8			
Absent	13.1			
				
*BM blasts*		0.9		
>15%	10			
⩽15%	11.3			
				
*Transfusions ⩾4 per month*		0.029		0.01
Yes	7.2		2.16 (1.2–4)	
No	11		1	

Abbreviations: ANC, absolute neutrophil count; BM, bone marrow; CI, confidence interval; HR, hazard ratio; IPSS-R, revised international prognostic scoring system; OS, overall survival; PB, peripheral blood; WPSS, WHO classification-based prognostic scoring system.
