# Supplementary material for: Expression of CD25 antigen on CD34+ cells is an independent predictor of outcome in late-stage MDS patients treated with azacitidine
Source: Blood Cancer J. 2014 Feb 28;4(2):e187–. doi: 10.1038/bcj.2014.9 (PMC3944665; doi:10.1038/bcj.2014.9)
Supplement: Supplementary Table S1 [file bcj20149x1.doc]

|  | **CD25- (n=36)** | **CD25+ (n=25)** | **p-value** |
| --- | --- | --- | --- |
| **Age** | 72.5 (53.4-83.5) | 72.9 (52-81.7) | 0.2 |
| >65 | 31 (86%) | 17 (68%) |  |
| <65 | 5 (14%) | 8 (32%) |  |
| **Sex** |  |  | 0.027 |
| Male | 20 (55%) | 21 (84%) |  |
| Female | 16 (45%) | 4 (16%) |  |
| **Baseline blood counts** |  |  |  |
| Hemoglobin (g/dl) | 8.6 (6.1-10.6) | 8.8 (6.8-11.5) | 0.78 |
| ANC(x 10^9^/L) | 1.1 (0.04-13.4) | 2.8 (0.08-23) | 0.07 |
| Platelets (x 10^9^/L) | 66 (9-383) | 50 (11-181) | 0.42 |
| **Number of completed cycles** |  |  | 0.14 |
| Median (range) | 6 (1-37) | 5 (1-33) |  |
| **WHO classification** |  |  | 0.28 |
| RCMD | 2 (6%) | 0 (0%) |  |
| RAEB-I | 0 (0%) | 1 (4%) |  |
| RAEB-II | 17 (47%) | 13 (52%) |  |
| CMML-II | 6 (16%) | 7 (28%) |  |
| AML-MDS | 11 (31%) | 4 (16%) |  |
| **IPSS** |  |  | 0.7 |
| Intermediate-2 | 18 (50%) | 9 (36%) |  |
| High | 17 (47%) | 11 (44%) |  |
| N/A | 1 | 5 |  |
| **WPSS** |  |  | 0.22 |
| High | 15 (42%) | 8 (32%) |  |
| Very high | 6 (16%) | 7 (28%) |  |
| N/A | 15 (42%) | 10 (40%) |  |
| **IPSS-R** |  |  | 0.8 |
| Intermediate | 3 (8%) | 1 (4%) |  |
| High | 14 (39%) | 8 (32%) |  |
| Very high | 18 (50%) | 12 (48%) |  |
| N/A | 1 | 4 |  |
| **IPSS-R Cytogenetic risk** |  |  | 0.72 |
| Good | 18 (50%) | 10 (40%) |  |
| Intermediate | 8 (22%) | 6 (24%) |  |
| Poor | 5 (14%) | 6 (24%) |  |
| Very poor | 4 (11%) | 2 (8%) |  |
| N/A | 1 | 1 |  |
| **PB blasts** |  |  | 0.6 |
| Present | 21 (58%) | 14 (56%) |  |
| Absent | 15 (42%) | 11 (44%) |  |
| **BM blasts** |  |  | 0.2 |
| >15% | 18 (50%) | 9 (36%) |  |
| ≤15% | 18 (50%) | 16 (64%) |  |
| **Transfusions ≥ 4 per month** |  |  | 0.48 |
| Yes | 23 (64%) | 17 (68%) |  |
| No | 13 (36%) | 8 (32%) |  |
| **Treatment after**  **azacitidine failure** |  |  |  |
| Intensive chemotherapy | 0 | 2 |  |
| Allo-SCT | 2 | 0 |  |
| **Response** |  |  | 0.4 |
| CR | 12 (33%) | 4 (16%) |  |
| Hematologic improvement | 5 (14%) | 4 (16%) |  |
| Stable disease | 8 (22%) | 5 (20%) |  |
| Failure | 11 (31%) | 12 (48%) |  |

**Supplementary Table 1.** Baseline patient characteristics (n=61); N/A: not applicable/not available; CR: complete response.
