# Supplementary material for: Expression of CD25 antigen on CD34+ cells is an independent predictor of outcome in late-stage MDS patients treated with azacitidine
Source: Blood Cancer J. 2014 Feb 28;4(2):e187–. doi: 10.1038/bcj.2014.9 (PMC3944665; doi:10.1038/bcj.2014.9)
Supplement: Supplementary Table S2 [file bcj20149x2.doc]

|  | Univariate analysis | | Multivariate analysis | |
| --- | --- | --- | --- | --- |
|  | Median EFS | p-value | HR (95% CI) | p-value |
| Age |  | 0.70 |  |  |
| >65 | 9.0 |  |  |  |
| <65 | 9.3 |  |  |  |
| Sex |  | 0.84 |  |  |
| Male | 10 |  |  |  |
| Female | 8.6 |  |  |  |
| CD25 status |  | 0.023 |  | 0.008 |
| Positive (>20%) | 6.7 |  | 2.2 (1.23-3.9) |  |
| Negative (<20%) | 13.6 |  | 1 |  |
| ANC(x 10^9^/L) |  | 0.72 |  |  |
| ≥ 1 | 9.9 |  |  |  |
| < 1 | 8.5 |  |  |  |
| Platelets (x 10^9^/L) |  | 0.33 |  |  |
| ≥ 100 | 10.4 |  |  |  |
| < 100 | 8.6 |  |  |  |
| IPSS |  | 0.92 |  |  |
| Intermediate-2 | 9.0 |  |  |  |
| High | 8.6 |  |  |  |
| WPSS |  | 0.72 |  |  |
| High | 9.0 |  |  |  |
| Very high | 6.7 |  |  |  |
| IPSS-R |  | 0.5 |  |  |
| Intermediate | 6.2 |  |  |  |
| High | 10.0 |  |  |  |
| Very high | 6.7 |  |  |  |
| IPSS-R Cytogenetic risk |  | 0.61 |  |  |
| Good | 9.9 |  |  |  |
| Intermediate | 6.4 |  |  |  |
| Poor | 9.3 |  |  |  |
| Very poor | 6.7 |  |  |  |
| PB blasts |  | 0.68 |  |  |
| Present | 6.7 |  |  |  |
| Absent | 10.4 |  |  |  |
| BM blasts |  | 0.72 |  |  |
| >15% | 9 |  |  |  |
| ≤15% | 10 |  |  |  |
| Transfusions ≥ 4 per month |  | 0.028 |  | 0.008 |
| Yes | 6.3 |  | 2.23 (1.23-4) |  |
| No | 10.4 |  | 1 |  |

Table S2. Prognostic factors for event-free survival.

|  |  |  |  |
| --- | --- | --- | --- |
