# Supplementary material for: Expression of CD25 antigen on CD34+ cells is an independent predictor of outcome in late-stage MDS patients treated with azacitidine
Source: Blood Cancer J. 2014 Feb 28;4(2):e187–. doi: 10.1038/bcj.2014.9 (PMC3944665; doi:10.1038/bcj.2014.9)
Supplement: Supplementary Figure S1 [file bcj20149x3.doc]

**
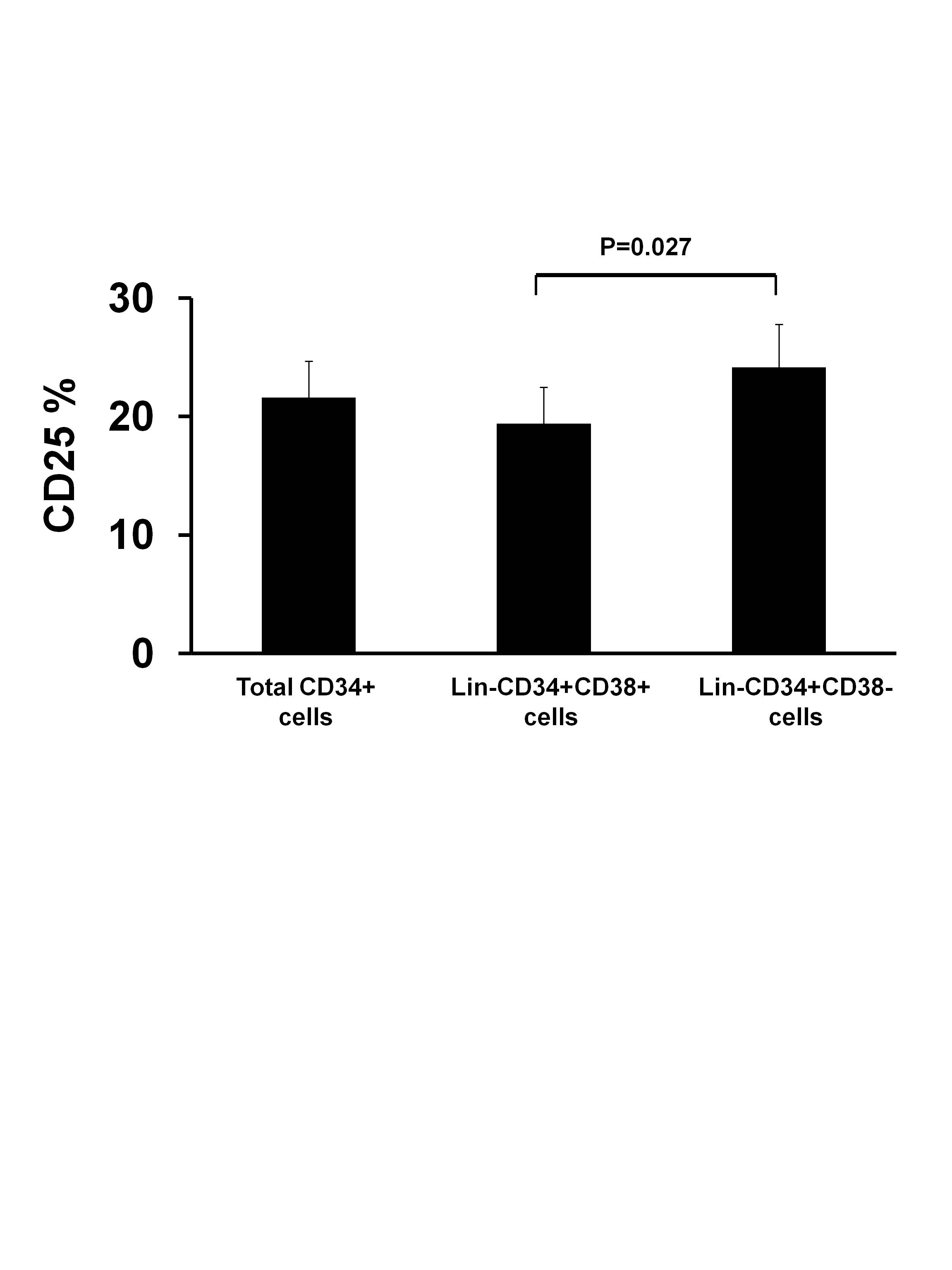
**

**Figure S1. Expression of CD25 in various hematopoietic compartments of CD34+ progenitors**

Significantly higher pretreatment levels of CD25 were noted on Lin^-^CD34^+^CD38^-^ cells, a subpopulation enriched in Leukemia propagating cells. Data are presented as mean ± standard error of the mean (SEM) and the significance of the differences was assessed by repeated measures ANOVA.
