# Supplementary material for: Expression of CD25 antigen on CD34+ cells is an independent predictor of outcome in late-stage MDS patients treated with azacitidine
Source: Blood Cancer J. 2014 Feb 28;4(2):e187–. doi: 10.1038/bcj.2014.9 (PMC3944665; doi:10.1038/bcj.2014.9)
Supplement: Supplementary Figure S2 [file bcj20149x4.doc]

**
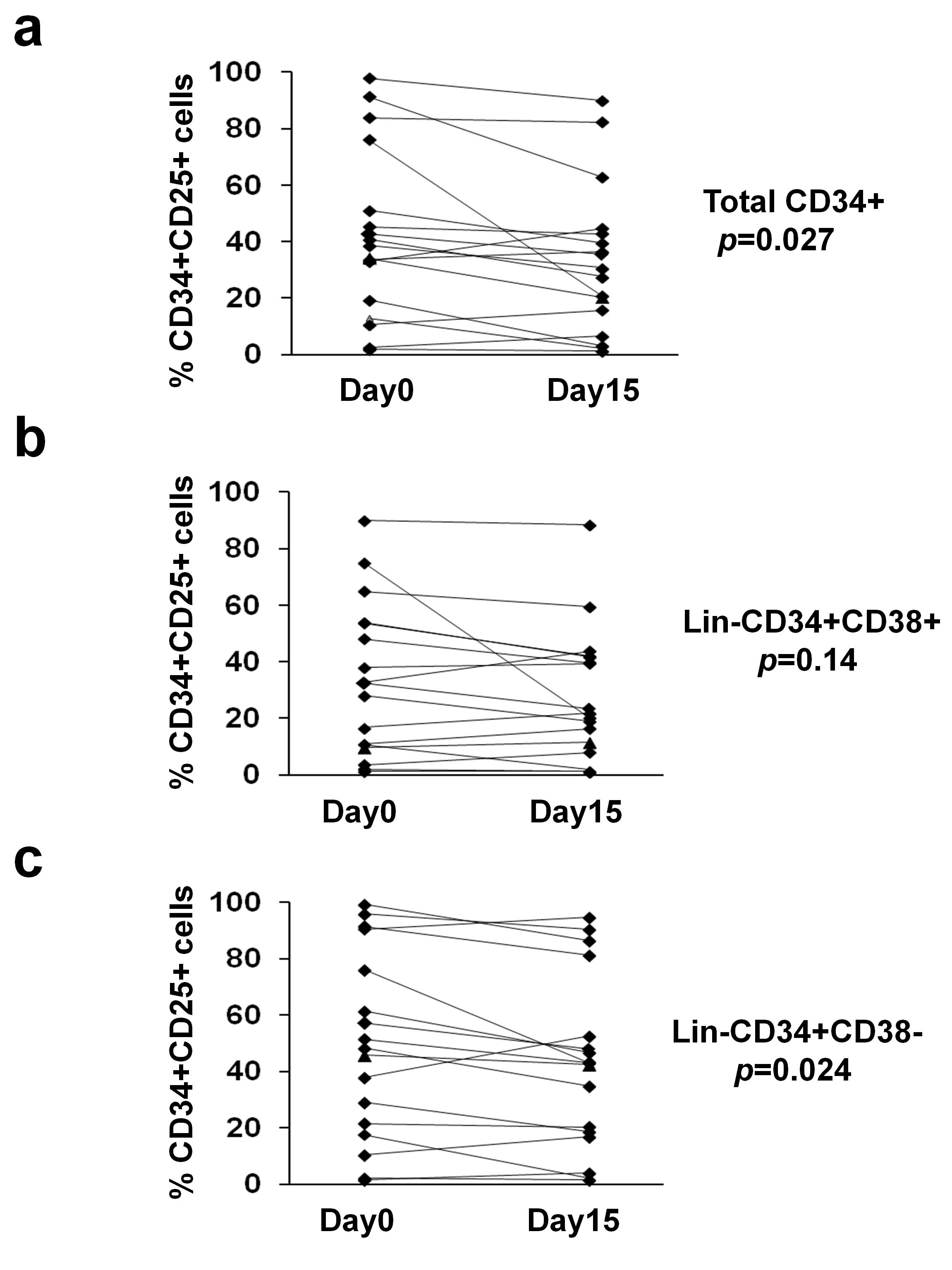
**

**Figure S2. Differential epigenetic modulation of CD25 expression in the various hematopoietic compartments of CD34+ progenitors.**

CD25 was significantly downregulated after azacytidine treatment on total CD34+ cells (a). However, this reduction was due exclusively to the marked downregulation of CD25 in the LSC compartment (c), whereas CD25 expression remained stable in committed progenitors of the same patients (b).
