# Supplementary material for: Expression of CD25 antigen on CD34+ cells is an independent predictor of outcome in late-stage MDS patients treated with azacitidine
Source: Blood Cancer J. 2014 Feb 28;4(2):e187–. doi: 10.1038/bcj.2014.9 (PMC3944665; doi:10.1038/bcj.2014.9)
Supplement: Supplementary Figure S3 [file bcj20149x5.doc]

**
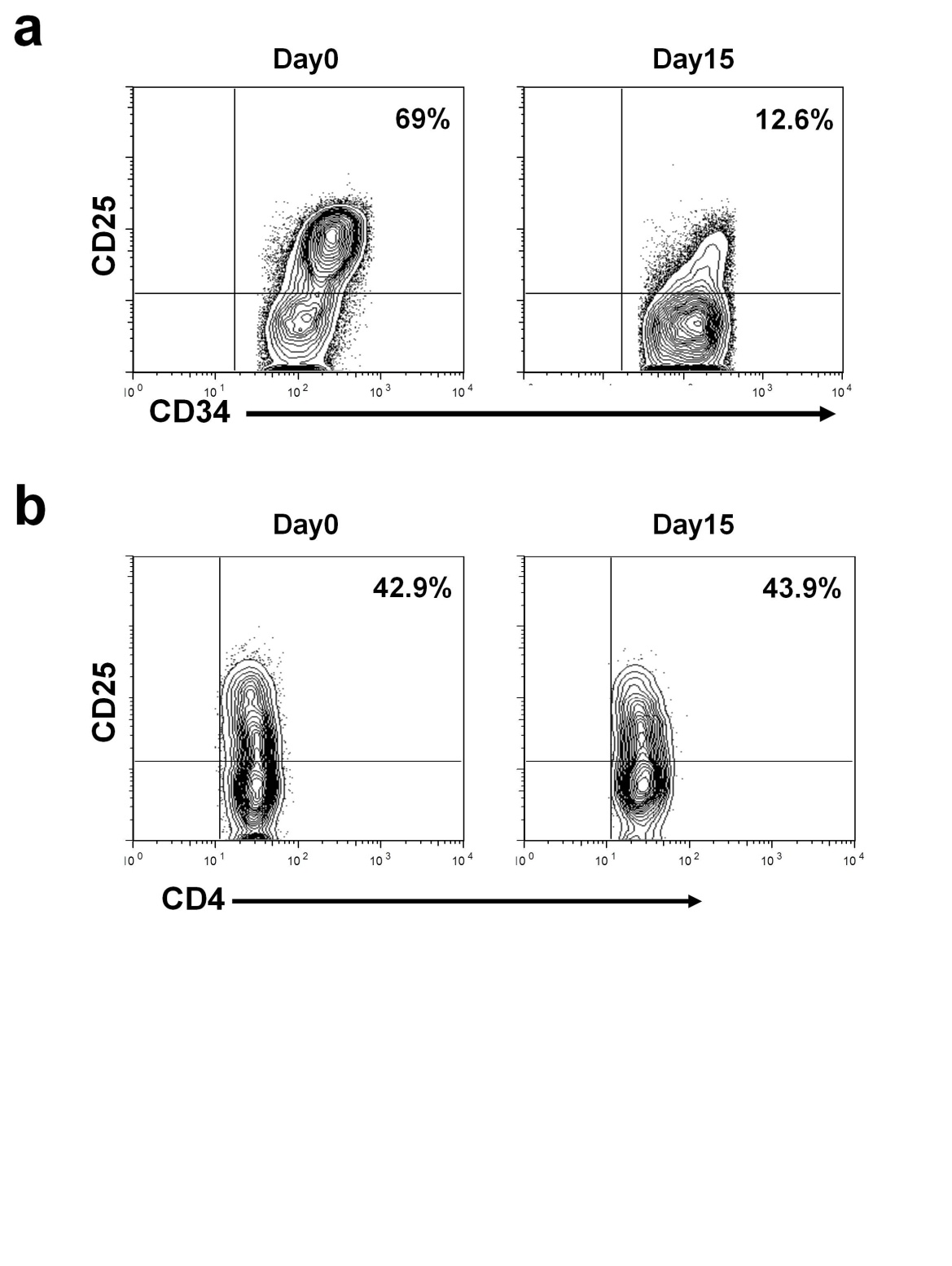
**

**Figure S3. Kinetics of CD25 expression after azacytidine administration in distinct cell types.**

CD25 was significantly downregulated after azacytidine treatment on total CD34+ cells (a), whereas CD4+ cells from the same patient retained comparable levels and expression pattern of CD25 (b).
