# Supplementary material for: Expression of CD25 antigen on CD34+ cells is an independent predictor of outcome in late-stage MDS patients treated with azacitidine
Source: Blood Cancer J. 2014 Feb 28;4(2):e187–. doi: 10.1038/bcj.2014.9 (PMC3944665; doi:10.1038/bcj.2014.9)
Supplement: Supplementary Materials and Methods [file bcj20149x6.doc]

**Supplementary Materials and Methods**

**Antibodies, data acquisition and analysis of CD25 expression**

Mononuclear bone marrow cells were isolated after density centrifugation and stained with the following antibodies: CD25 (2A3), CD34 (8G12), CD38 (HB7) and the appropriate isotype controls, all from BD Biosciences. To stain for lineage positive and negative cells we used an antibody cocktail consisting of the following antibodies: CD4 (RPA-T4), CD2 (clone RPA-2.10), CD3 (HIT3a), CD8 ( RPA-T8), CD19 (HIB19), CD20 (2H7) and GPA (GA-R2) as previously reported. Data were acquired on a 4-color FACSCalibur (BD Biosciences, CA, USA) cytometer and analyses were done by using Flowjo software (Treestar, Ashland, OR).

## Supplementary Information References

1. Goardon N, Marchi E, Atzberger A, Quek L, Schuh A, Soneji S*, et al.* Coexistence of LMPP-like and GMP-like leukemia stem cells in acute myeloid leukemia. *Cancer cell* 2011 Jan 18; **19**(1)**:** 138-152.
